# Multi-Scale Feature Learning Convolutional Neural Network for Image Denoising

**DOI:** 10.3390/s23187713

**Published:** 2023-09-06

**Authors:** Shuo Zhang, Chunyu Liu, Yuxin Zhang, Shuai Liu, Xun Wang

**Affiliations:** 1 Changchun Institute of Optics, Fine Mechanics and Physics, Chinese Academy of Sciences, Changchun 130033, China; zhangshuo21a@mails.ucas.ac.cn (S.Z.); zhangyuxin@ciomp.ac.cn (Y.Z.); liushuai@ciomp.ac.cn (S.L.); wangxun@ciomp.ac.cn (X.W.); 2 University of Chinese Academy of Sciences, Beijing 100039, China; 3 Key Laboratory of Space-Based Dynamic & Rapid Optical Imaging Technology, Chinese Academy of Sciences, Changchun 130033, China

**Keywords:** multi-scale feature learning, denoising algorithm, convolutional neural network

## Abstract

Affected by the hardware conditions and environment of imaging, images generally have serious noise. The presence of noise diminishes the image quality and compromises its effectiveness in real-world applications. Therefore, in real-world applications, reducing image noise and improving image quality are essential. Although current denoising algorithms can somewhat reduce noise, the process of noise removal may result in the loss of intricate details and adversely impact the overall image quality. Hence, to enhance the effectiveness of image denoising while preserving the intricate details of the image, this article presents a multi-scale feature learning convolutional neural network denoising algorithm (MSFLNet), which consists of three feature learning (FL) modules, a reconstruction generation module (RG), and a residual connection. The three FL modules help the algorithm learn the feature information of the image and improve the efficiency of denoising. The residual connection moves the shallow information that the model has learned to the deep layer, and RG helps the algorithm in image reconstruction and creation. Finally, our research indicates that our denoising method is effective.

## 1. Introduction

Because of the impact of hardware devices and their surrounding conditions, noise will inevitably be generated during image transmission, which could potentially degrade the image quality. Denoising of images is a low-level vision task and an essential step for high-level vision tasks. Denoising of images holds a crucial significance in the domains of satellite remote sensing, medicine, military, and internet technology [1,2]. Mathematically, an image denoising model can be expressed as y = x + n, where y represents the original image, x corresponds to a noise-free clean image, and n represents the noise component.

Algorithms for image denoising can be broadly classified into three categories: filter-based approaches, learning-based techniques, and model-based methods. The filter-based approach employs a few manually created filters to eliminate image noise. The adaptive Wiener filter [3], the bilateral filter, the Gaussian filter, and the median filter [4] are some of the more well-known filter-based algorithms. Nevertheless, these algorithms require manual parameter tuning, and there is a risk of losing image details during the denoising process [5,6,7,8,9].

For model-based techniques, the distribution of the images and noise must be modeled. The technique is then optimized while attempting to generate a clear image using the distribution of the model as a prior. As a result, the model-based algorithm’s first phase entails capturing the noise characteristics that are built into the image and then using what is already known about the image to remove the noise in an efficient manner. The non-local mean (NLM) algorithm utilizes a weighted average of blocks that share similarities with each other in order to eliminate noise [10]. The BM3D algorithm realizes image denoising by enhancing sparsity [8]. Different from the general low-rank clustering algorithm, WNNM [11] utilizes distinct weights for singular values to maximize the utilization of prior knowledge. This approach involves leveraging prior information to determine the kernel norm employed in the process of image denoising. Finally, the effect of denoising is obtained. However, the shortcomings of these algorithms are also obvious. The level of noise must be identified in advance, and the denoising process in the testing phase is time-consuming due to the algorithm’s intricate optimization problems. This complexity leads to a prolonged duration for achieving optimal denoising results. In an effort to enhance the denoising capabilities, the CSF algorithm uses the statistical characteristics of the model based on random fields and the optimization ability of the expanded semi-quadratic algorithm to reduce the noise [12]. By performing a predetermined number of gradient descent iterations, the TNRD algorithm [13] can progressively update the denoised image, iteratively reduce noise and enhance image quality. While both CSF and TNRD algorithms exhibit their own unique strengths, they are essentially limited to fixed priors, and these algorithms are specific to specific noise, so their processing on blind noise is not ideal.

Thanks to AlexNet [14], ResNet [15], and other models, the denoising algorithm based on learning is very effective in processing images, and the image denoising algorithm based on convolutional neural network (CNN) has demonstrated remarkable performance and achieved significant advancements in the field [16,17,18,19,20,21]. For instance, a feed-forward denoising convolutional neural network (DnCNN [22]), which combines the principles of residual learning and batch normalization, designs an end-to-end network. The algorithm learns the noise of noisy pictures and then effectively improves the effect of denoising. Zhang et al. introduce an innovative denoising algorithm that is characterized by its speed and flexibility (FFDNet) [23]. Tian et al. propose an algorithm that uses residual learning and BN to solve model training difficulties (ECNDNet [24]). In order to extract more image information, the algorithm uses dilated convolution to extract context information. Tian et al. propose an algorithm that increases the influence of shallow features on deep features and propose four modules (ADNet [25]). Tian et al. propose an algorithm (BRDNet [26]) that combines two networks to increase the network width. Kligvasser et al. propose a denoising algorithm (xUnit [27]) using a new activation function, which reduces the parameters of the model as much as possible while ensuring the effect of the algorithm remains unchanged. Although these denoising techniques have successfully reduced noise, it is important to acknowledge that their feature extraction methods rely on fixed-scale approaches. This limitation restricts their ability to fully extract and utilize the rich information present in the image. Gou et al. introduce a noteworthy improvement in the field of image denoising with their proposed multi-scale adaptive network (MSANet [28]), which considers both the characteristics of the scale and the complementarity across scales and integrates them into the multi-scale design, which effectively improves the denoising performance of the image. However, the algorithm still does not take into account the loss of image details.

Building upon the aforementioned challenges, this paper introduces an innovative denoising algorithm based on the FL module and RG module. The algorithm improves the overall denoising process by transferring shallow information to the deeper layers of the network. The FL module can fully utilize the Res2Net module to extract image features [29], the information of the image is extracted from the perspectives of different dimensions, and detailed information is preserved. The residual connection transfers the shallow information to the deep network, helps the algorithm combine global and local information, improves the effect of an algorithm, and reduces the complexity of a model.

The main contributions of this paper are as follows:

(1) This algorithm uses the Res2Net network structure to design the FL module and the RG module. The FL module fully extracts image information from different scales, uses RG to reconstruct a clean image, improves the denoising performance of the algorithm, and ensures that detailed information in the image is preserved without being lost.

(2) This paper incorporates residual connections, enabling the transfer of information from shallow layers to deep layers. This combination of global and local features enhances the noise reduction efficiency of the algorithm. Additionally, this approach helps reduce the complexity of the model, making it more computationally efficient.

(3) This paper presents experimental results on datasets to validate the proposed approach for image denoising. The results demonstrate that MSFLNet achieves good performance in terms of denoising quality, as evidenced by excellent values of peak signal-to-noise ratio (PSNR) and structural similarity index (SSIM).

The remaining parts of this article are as follows. Section 2 discusses the relevant existing work related to the proposed method. Section 3 details the proposed method. It presents the algorithm, network architecture, and techniques used in the study. In Section 4, this article introduces a large number of experimental results generated using the proposed method. This paper concludes with Section 5, which summarizes the key findings, contributions, and implications of the research.

## 2. Related Work

### 2.1. Residual Connection

As the number of network layers increases, the algorithm’s effectiveness can indeed be improved to a certain extent, but one problem that can arise with deeper networks is the gradient explosion. To overcome the challenge and improve the algorithm’s performance, the residual block that ResNet proposes combines the input of the original image with the output of several layers and feeds it to the following layer. The incorporation of residual connections plays a vital role in enabling the transfer of information from the shallow layer to the deep network within the algorithm, which can help the algorithm combine local and global. It can also solve a series of problems arising from the increase in the number of network layers.

### 2.2. Res2Net

**Figure 1:**
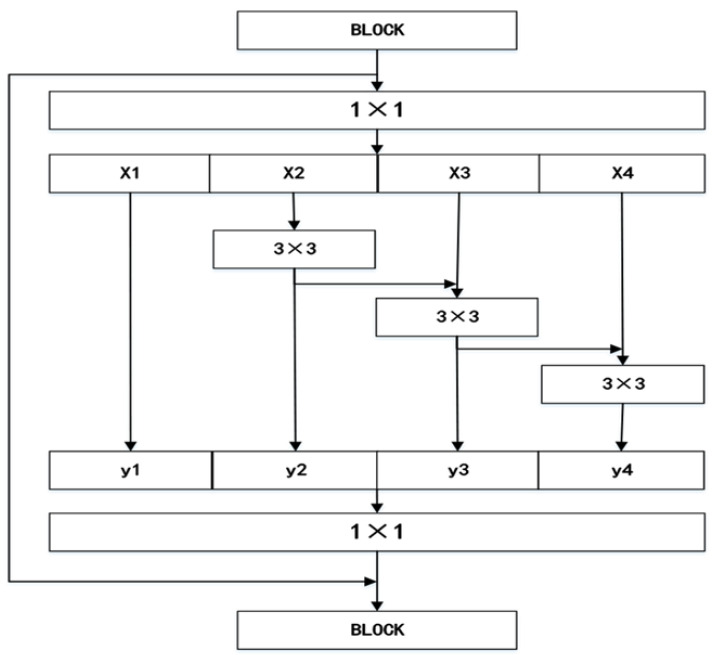
Res2Net module. Res2Net proposes a multi-scale module built inside residual blocks to form receptive fields of different sizes and obtain different fine-grained features.

Multi-scale feature learning methods differ from fixed-scale extraction methods. The multi-scale module excels at extracting image information from diverse dimensions, improving the efficiency of image denoising. Based on this, Res2Net proposes a multi-scale module built inside the residual block to form receptive fields of different sizes and obtain different fine-grained features. As shown in Figure 1, after the image information passes through the 1 × 1 convolutional layer, the image’s feature information is segmented into s subsets, where s is the number of subsets into which the feature information is divided. The segmented image information is represented by $x_{i}$, where every part is the same size. But the number of channels is 1/s of the input feature map of the previous layer, where each part has a corresponding 3 × 3 convolution, $h_{i}\left(x_{i}\right)$ represents the 3 × 3 convolution, and $y_{i}$ represents the output of multi-scale feature learning methods. Each part is fused with each other after passing through different convolutional layers, and finally, the network will learn image information from different scale dimensions. As shown in Formula (1), the information of the image is represented by $x_{i}$. Further information is learned and represented by $y_{i}$ after Res2Net extracts the image information $x_{i}$.

(1) $$\begin{array}{cc} y_{i}=\left\{\begin{array}{c} x_{i}\\ h_{i}\left(x_{i}\right)\\ h_{i}\left(x_{i}+y_{i-1}\right) \end{array}\right. & \end{array}\begin{array}{c} i=1\\ i=2\\ 2<i\leq s \end{array}$$

## 3. Network Structure

In this section, the algorithm will be introduced, which is composed of three FL modules, one RG module, and a residual connection. The FL module makes full use of the learning method of multi-scale features to obtain image information. The multi-scale feature learning method extracts noise and details from different dimensions of the image. The RG module utilizes the image information learned by the FL module to reconstruct and generate clean images.

### 3.1. MSFLNet Module

**Figure 2:**
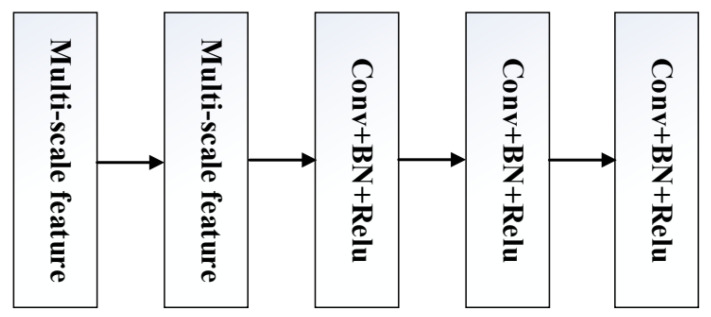
FL module. The FL module is composed of two multi-scale feature modules (Res2Net) and three ResNetConv+BN+ReLu.

The network structure of MSFLNet is visually represented in Figure 2. First, the noise-containing picture is input to the Conv layer of the first layer, the information of the image is initially extracted, and then all the information is passed to three FL modules and one RG module. The FL module contains two multi-scale feature modules (Res2Net) and three Conv+BN+ReLu (convolutional layer + batch normalization layer + activation function). The model is too deep in the network training process, which may cause the algorithm to produce a gradient explosion, and the gradient explosion will affect the effect of the algorithm. Therefore, to address the issue of gradient explosion, expedite network convergence, and facilitate easier training, the MSFLNet architecture incorporates batch normalization (BN) layers. These BN layers normalize the data flowing through the convolutional (Conv) layers. This normalization process ensures that the data are centered and scaled, preventing the gradients from becoming excessively large or small during training. By maintaining stable gradients, BN accelerates the convergence of the network and aids in more efficient training of the algorithm. By incorporating ReLU activation, MSFLNet can effectively capture and represent complex and non-linear relationships within the data. This is achieved by enabling the network to learn and propagate both positive and negative activations, allowing for richer feature representation and increased expressive power. The FL module fully utilizes multi-scale feature learning methods of Res2Net to extract more feature information. But unlike the three FL modules, the RG module is composed of two multi-scale feature modules (Res2Net), two Conv+BN+ReLu (convolution layer + batch normalization layer + activation function), one Conv+BN, and one Conv, and the RG module helps the algorithm to reconstruct and generate a clean image. By combining the original information of the image with the information propagated through the second FL module, the residual connection establishes a direct pathway for information transfer.

### 3.2. FL Module

In image denoising algorithms, the key challenge lies in effectively extracting the noise from the image while preserving the essential information of the clean image. Therefore, to ensure that an algorithm effectively removes noise from an image while retaining the complete information from the original image, the algorithm designs an FL feature extraction module. The FL module for extracting features is composed of two multi-scale feature modules (Res2Net) and three Conv+BN+ReLu (convolution layer + batch normalization + activation function) modules. Two multi-scale extraction modules are connected together that can leverage the inherent characteristics of the multi-scale feature learning methods, extract image information from different dimensions, and add three ResNetConv+BN+ReLu after the two multi-scale feature learning methods, which can improve the extraction ability of the FL module. In ResNetConv+BN+ReLu (convolution layer + batch normalization + activation function), the function of the convolution layer is to extract image feature information. BN can perform batch normalization operations on the feature information extracted by the convolution layer, which can significantly expedite the convergence of the network to mitigate the issue of gradient explosion. The ReLu activation function can help the model provide non-linear capabilities and accelerate the training of the network model. Therefore, adding three ResNetConv+BN+ReLu (convolutional layer + batch normalization + activation function) methods after the two multi-scale feature learning methods to form the FL module can effectively enhance the network’s feature extraction capability. Assuming that the information of the first 3 × 3 convolutional layer of the model is passed to the FL module, the first multi-scale module first extracts the image information, as shown in Equation (2); *X* denotes the image information; while R1 signifies the output of the first multi-scale module, namely:

(2) $$R1=R(Conv_{3\times 3}\left(X\right))$$

Then, after the first multi-scale module learns the image information, it transfers the information to the second multi-scale module. R2 is represented as the output of the second multi-scale module, namely:

(3) R2=R(R1(X))

After the second multi-scale module learns the image information, the information is first passed to the first ResNetConv+BN+ReLu (convolution layer + batch normalization + activation function). CBR is expressed as the output of ResNetConv+BN+ReLu, namely:

(4) CBR=Relu(BN(Conv3×3(R2(R1(X)))))

The information of the image is then transmitted to the second and third layers of ResNetConv+BN+ReLu (convolution layer + batch normalization + activation function), and finally, FL outputs information, represented by REC. $CBR_{1}$, $CBR_{2}$, and $CBR_{3}$ represent the output of ResNetConv+BN+ReLu (convolution layer + batch standardization + activation function) of the first, second, and third layers, respectively, namely:

(5) $$REC=CBR_{3}\left(CBR_{2}\left(CBR_{1}\left(R2\left(R1\left(X\right)\right)\right)\right)\right))$$

### 3.3. RG Module

**Figure 3:**
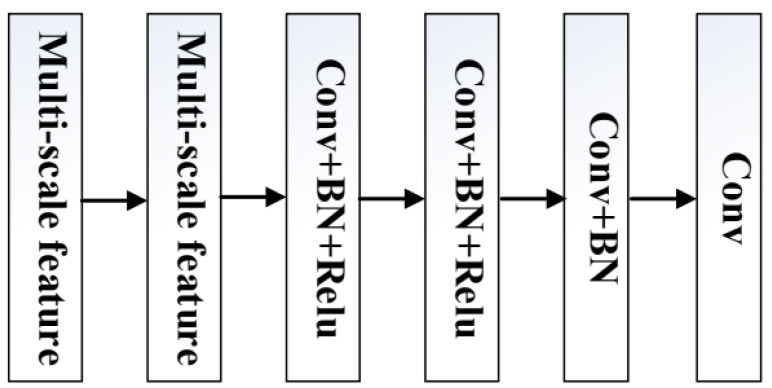
RG module. RG module is composed of two multi-scale feature modules (Res2Net), two ResNetConv+BN+ReLu (convolution layer + batch normalization layer + activation function) modules, one Conv+BN module, and one Conv module.

After all the image information is learned by the network model, it needs to be reconstructed to generate a clean image. Therefore, we designed an RG module for image reconstruction, as shown in Figure 3, which consists of two multi-scale feature modules (Res2Net), two ResNetConv+BN+ReLu (convolution layer + batch normalization layer + activation function) modules, one Conv+BN module, and one Conv module. Two multi-scale feature modules (Res2Net) can help the algorithm extract the image information learned by the network and ultimately transfer all information to the last Conv layer to generate a clean image.

After the last FL module learns the image information, it transfers all the information to the RG module and reconstructs and generates a clean image, where R3 represents the result of the first multi-scale feature module (Res2Net) in the RG module, R4 represents the result of the second multi-scale feature module (Res2Net), and x is the result of the previous module. $CBR_{4}$ and $CBR_{5}$ represent the output of ResNetConv+BN+ReLu (convolution layer + batch standardization + activation function) of the first and second layers, respectively. CB represents the output of Conv+BN (convolutional layer + batch normalization), C2 represents the input of the last layer of Conv, and C3 represents the output of the RG module, namely:

(6) $$C2=CB(CBR_{5}\left(CBR_{4}\left(R4\left(R3\left(x\right)\right)\right)\right))$$

(7) $$C3=Conv_{3\times 3}\left(C2\right)$$

### 3.4. Loss Functions and Optimizers

The convolutional neural network utilizes the loss function to quantify the disparity between the actual value and the predicted value. A smaller loss function indicates a superior performance of the algorithm. The smooth curve of the mean squared error (MSE) loss function facilitates network training. Hence, this algorithm adopts the MSE loss function, which is also referred to as the L2 loss function. As shown in Equation (8), N represents the total number of images in the training set, $x_{i}$ represents the image obtained from training the neural network with noisy images, and $y_{i}$ represents the clean image corresponding to the noisy images.

(8) $$MSE=\frac{1}{N}\sum_{i=1}^{N}{\left(x_{i}-y_{i}\right)}^{2}$$

Throughout the model training process, the optimizer plays a crucial role in facilitating parameter updates and guiding the model towards its optimal state. By combining the strengths of AdaGrad (adaptive gradient) and RMSProp (root mean square prop), the Adam optimizer leverages the advantages of both optimization algorithms. Taking into account a comprehensive estimation of the first-order and second-order moments of the gradient, the Adam optimizer calculates the update step size. The Adam optimizer is simple to implement and takes up less memory. It is particularly well suited for models with large-scale data and parameters. Hence, this article chooses Adam to help the model train to the optimal solution.

## 4. Experimental Results and Analysis

In this section, we will introduce the experiments of the algorithm on several image test sets, and conduct quantitative and qualitative analysis of the experimental settings and experimental results.

### 4.1. Experimental Environment

In order to give full play to the effect of our model, the learning rate is initially set to 0.0001, which is reduced to the original 0.2 every 30 epochs. During the training process, the batch size is set to 128, the patch is set to 40 × 40, and the Adam optimizer is selected. The training of this algorithm is conducted within a deep learning environment based on PyTorch 1.11.0 and Python 3.8 on an Ubuntu 20.04 system. The GPU is NVIDIA GeForce RTX3080, and cuda11.3 and conda8.2.1 are used to accelerate the network training of the GPU.

### 4.2. Training Dataset

The data sets used by the algorithm are Train400 [23], DIV2K [30], and SIDD. Train400 is 400 pictures in the Berkeley segmentation data set. The data set contains 400 clear grayscale pictures of 180 × 180. The pictures are rich in content, including various types of animals, landscapes, faces, and more. To improve the denoising performance of our algorithm, 800 pictures in the DIV2K dataset are selected as part of the dataset. The DIV2K dataset is a relatively common dataset in the field of super-resolution reconstruction. In order to facilitate training, it is scaled to a 180 × 180 size picture, and the data set is expanded by flipping the data set by 90°, 180°, 270°, and zooming. In order to train our MSFLNet algorithm model, the model trains Gaussian noise with noise levels of 15, 25, and 50, sets a patch size of 40 × 40, and finally, we generated 715,200 patches for image noise training. For real noise denoising, The algorithm selects the SIDD dataset. SIDD is a smartphone image denoising training set that includes paired clean and noisy images. We chose 140 images and cut them to 1024 × 1024 in size. We expanded the dataset by performing data augmentation on those images in order to increase the dataset.

### 4.3. Test Dataset

To validate the efficacy of our algorithm in removing noise, BSD68 [23] and Set12 [23] are selected. BSD68 contains 68 grayscale images with rich content, and Set12 is a dataset with 12 grayscale images. We conduct experiments on two test sets at noise levels of 15, 25, and 50. For the experiment on real noise images, we selected images from the SIDD dataset and PolyU dataset for the experiment. PolyU is a large-scale dataset containing real-world noisy images. We selected 14 images from the SIDD dataset and cropped them to 1024 × 1024 in size. Similarly, we selected 16 images from the PolyU dataset and cropped them to 1024 × 1024 in size. The algorithm selects the TNO dataset to test the denoising of infrared images. TNO is a dataset that integrates infrared and visible light images, including infrared and visible light images in military, security, and other scenarios. This algorithm cropped 19 images from the TNO dataset and tested the denoising of infrared images on them.

### 4.4. Experimental Analysis

We use DnCNN, xUnit, ECNDNet, ADNet, MSANet, and this algorithm to test on BSD68 and Set12. We first conduct experimental comparisons on the BSD68 test set. As shown in Table 1 and Table 2, our algorithm outperforms other algorithms in PSNR and SSIM on the BSD68 test.

As shown in Table 3, we experimented with the algorithm on the Set12 test set. redAs shown in Table 4, our algorithm exhibits higher SSIM indicators compared to other algorithms. We experimented with all the algorithms on each picture on Set12 and tested their PSNR values. As shown in Table 3, our algorithm performs better in denoising experiments with a noise level of 50, and also performs well in experiments with other noise levels.

**Figure 4:**
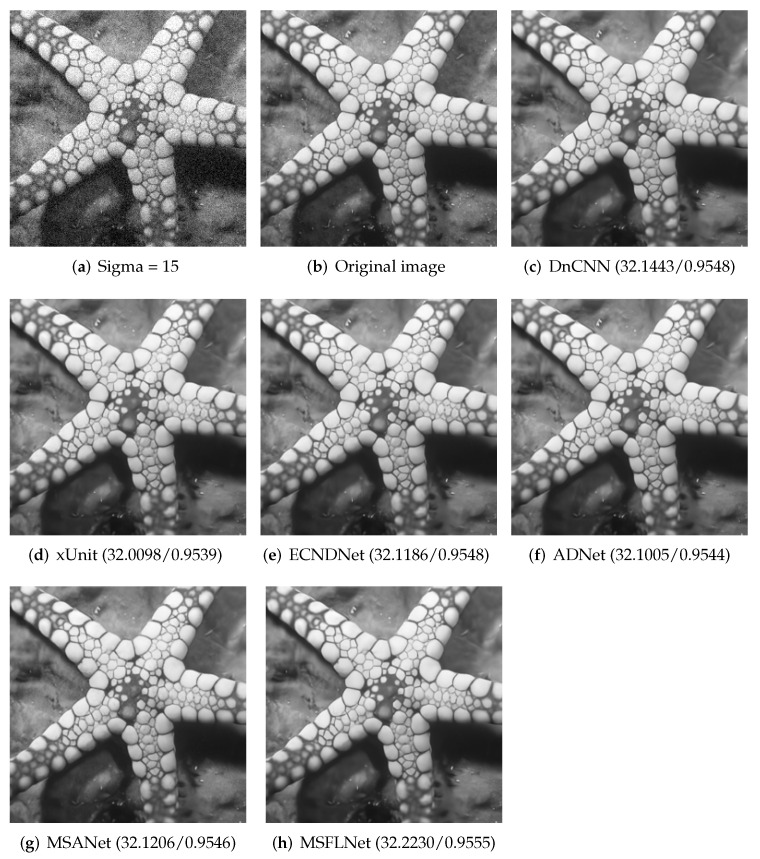
Results of selecting an image from the Set12 test set and denoising it with different algorithms when the noise level is 15.

**Figure 5:**
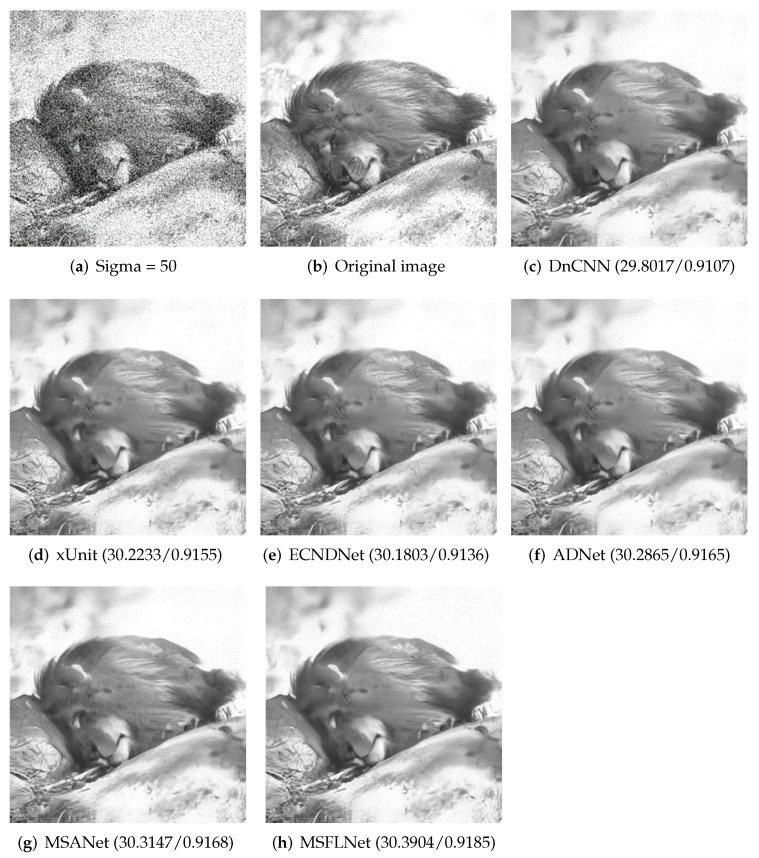
When the noise level is 50, the result of denoising an image selected from BSD68 with different algorithms.

We selected a picture in the BSD68 dataset and the Set12 dataset and provided a comparison of the denoising results between our algorithm and other algorithms. As shown in Figure 4 and Figure 5, the figure clearly demonstrates that our algorithm produces denoised results that are notably clearer while effectively preserving the details of the image. And the indicators of PSNR and SSIM are also higher.

For the experiment on infrared image denoising, we selected images from the TNO dataset for the experiment. We selected 19 images from the dataset and cropped them to 256 × 256 in size. We tested the denoising of infrared images on the test set using DnCNN, xUnit, ECNDNet, ADNet, MSANet, and our algorithm. As shown in Table 4, our algorithm performs well on PSNR and SSIM.

**Figure 6:**
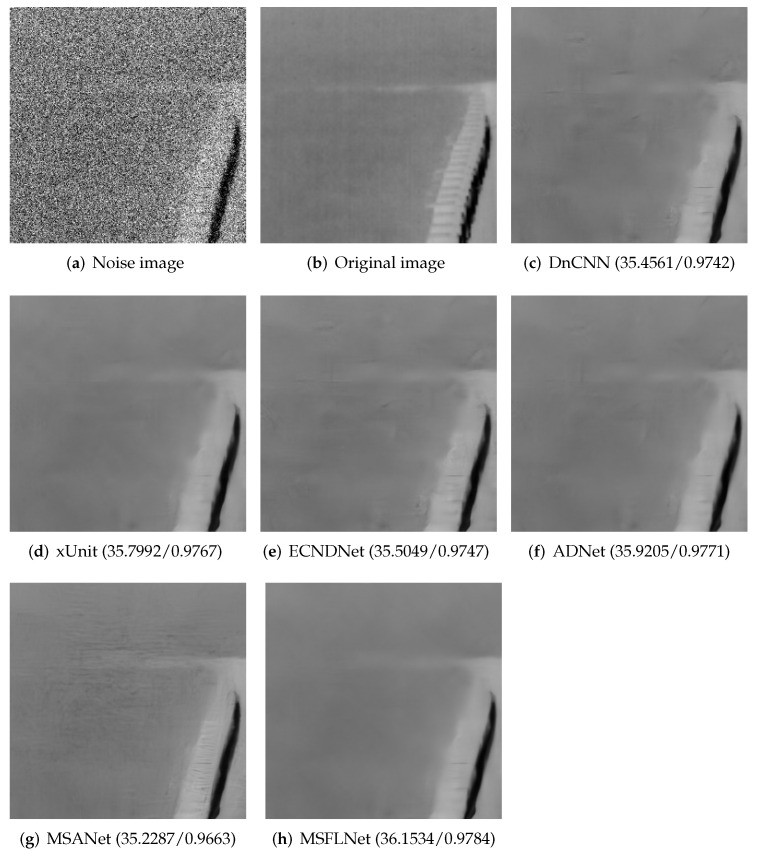
Results of selecting an image from the TNO test set and denoising it with different algorithms.

In the TNO dataset, we selected an image, and provided a comparison of the denoising results between our algorithm and other algorithms. As shown in Figure 6, the results show that our algorithm achieves clearer denoising results and preserves the details of the image.

We tested the denoising of real noisy images on SIDD and PolyU using DnCNN, xUnit, ECNDNet, ADNet, MSANet, and our algorithm. As shown in the Table 5 and Table 6, our algorithm performs well on PSNR and SSIM.

**Figure 7:**
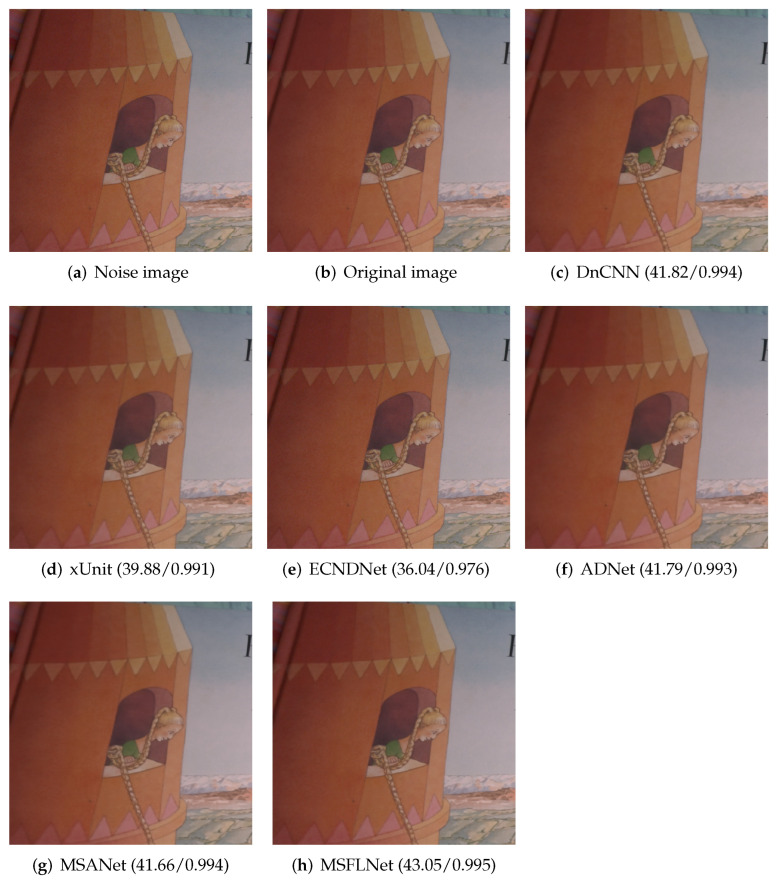
Results of selecting an image from the SIDD test set and denoising it with different algorithms.

We selected a picture in the SIDD dataset and listed the denoising results of our algorithm and other algorithms. As can be seen from the Figure 7, the denoising results of our algorithm are clearer, and the details of the picture are preserved.

### 4.5. Ablation Experiment

To verify the rationality of our algorithm, as shown in Table 7, we designed ablation experiments. On real noise images, we performed denoising experiments using ‘baseline model’, ‘RG+baseline’, ‘RG+baseline’, ‘RG+FL1’, ‘RG+FL2’, and ‘RG+FL’ (MSFLNet) in that order. The ‘baseline model’ represents replacing the model proposed by the algorithm with the same amount of convolutional layers. ‘RG+baseline’ and ‘FL+baseline’ denote the use of the corresponding blocks on the basis of the ‘baseline model’. Using RG modules on the base of the baseline module is indicated by the notation ’RG+baseline’. Meanwhile, using FL modules on the base of the baseline module is indicated by the notation ’FL+baseline’. On the basis of ’RG+baseline’, ’RG+FL1’ and ’RG+FL2’ indicate blocks employing one and two FL modules, respectively.

### 4.6. Ablation Experiment

The total number of model parameters (Parameters) and model computation (FLOPs) can reflect the complexity of the model to a certain extent. If the total number of model parameters and model computation are too large, the model is not suitable for practical applications. Therefore, in order to verify the rationality of the model, as shown in Table 8, we calculated the total number of model parameters and the amount of model calculations for each algorithm. From the table, it can be seen that the total number of parameters and calculation amount of our model are relatively reasonable. The model can effectively remove image noise in practical applications.

## 5. Conclusions

In this paper, we introduce a denoising algorithm that is built upon the MSFLNet network, which includes the three FL modules and the RG module we proposed. It uses the multi-scale feature extraction ability to extract image information from different dimensions and combines the image shallow information and deep information to help the network to learn image information, significantly enhance the denoising effectiveness, and improve the algorithm’s capability to preserve image details. The experiment proves the effectiveness of the MSFLNet algorithm in image denoising.

## Figures and Tables

**Table 1:** The average value of PSNR of different algorithms on the BSD68 test set at noise levels of 15, 25, and 50.

Data Set	Algorithm	Sigma = 15	Sigma = 25	Sigma = 50
BSD68	DnCNN	31.584	29.058	26.003
	xUnit	31.522	29.078	26.072
	ECNDNet	31.549	29.024	25.996
	ADNet	31.579	29.058	26.057
	MSANet	31.592	29.079	26.061
	MSFLNet	**31.594**	**29.096**	**26.139**

The bold one in the table is the best indicator.

**Table 2:** The average value of SSIM of different algorithms on the BSD68 test set at noise level 15, 25, and 50.

Data Set	Algorithm	Sigma = 15	Sigma = 25	Sigma = 50
BSD68	DnCNN	0.9416	0.9028	0.8265
	xUnit	0.9410	0.9035	0.8293
	ECNDNet	0.9414	0.9023	0.8268
	ADNet	0.9417	0.9027	0.8278
	MSANet	0.9420	0.9033	0.8285
	MSFLNet	**0.9420**	**0.9042**	**0.8314**

The bold one in the table is the best indicator.

**Table 3:** PSNR value and average value of each picture on Set12 for different algorithms.

Images	C.man	House	Peppers	Star.	Mon.	Air.	Parrot	Lena	Barbara	Boat	Man	Couple	Average
Noise Level							sigma = 15						
DnCNN	32.664	35.003	33.260	32.144	33.260	31.696	31.908	34.560	32.668	32.417	32.435	32.452	32.872
xUnit	32.524	34.894	33.165	32.009	33.108	31.634	31.869	34.467	32.422	32.359	32.389	32.361	32.767
ECNDNet	32.536	34.939	33.208	32.118	33.157	31.624	31.825	34.505	32435	32.401	32.408	32.393	32.796
ADNet	**32.7813**	**35.192**	**33.466**	32.100	33.247	31.790	31.979	**34.698**	**32.841**	**32.597**	32.473	32.578	**32.979**
MSANet	32.699	35.159	33.236	32.120	33.196	31.819	31.944	34.691	32.676	32.551	32.482	**32.578**	32.929
MSFLNet	32.777	35.137	33.408	**32.223**	**33.311**	**31.832**	**32.011**	34.638	32.765	32.497	**32.497**	32.560	32.972
Noise Level							sigma = 25						
DnCNN	30.264	33.138	30.810	29.394	30.455	29.087	29.444	32.422	30.057	30.217	30.085	30.091	30.455
xUnit	30.259	33.127	30.832	29.427	30.456	29.093	29,459	32.468	30.061	30.231	30.101	30.137	30.471
ECNDNet	30.138	33.009	30.764	29.361	30.374	29.041	29.419	32.356	29.902	30.171	30.056	30.023	30.385
ADNet	30.397	33.373	**31.077**	29.339	30.429	29.143	29.543	**32.624**	**30.316**	**30.388**	30.114	30.253	**30.583**
MSANet	30.251	**33.395**	30.936	**29.522**	30.409	29.148	29.424	32.604	30.236	30.340	**30.143**	**30.279**	30.557
MSFLNet	**30.428**	33.296	30.989	29.428	**30.522**	**29.183**	**29.606**	32.538	30.161	30.265	30.130	30.250	30.567
Noise Level							sigma = 50						
DnCNN	27.348	30.081	27.410	25.645	26.829	25.848	26.468	29.352	26.201	27.209	27.196	26.892	27.207
xUnit	27.362	30.171	27.438	25.716	26.906	25.859	26,396	29.479	26.298	27.268	27.233	27.001	27.261
ECNDNet	27.166	29.965	27.244	25.681	26.815	25,785	26.277	29.287	26.219	27.172	27.175	26.871	27.138
ADNet	27.410	30.417	27.603	25.685	26.888	25.866	26.642	29.606	26.563	**27.391**	27.237	27.088	27.366
MSANet	27.207	30.507	27.556	**26.019**	26.819	25,884	26,448	29.568	**26.841**	27.347	**27.292**	27.127	27.384
MSFLNet	**27.505**	**30.509**	**27.629**	25.85	**27.023**	**25.960**	**26.716**	**29.643**	26.809	27.38	27.284	**27.165**	**27.456**

The bold one in the table is the best indicator.

**Table 4:** The average value of PSNR and SSIM of different algorithms on the TNO test set at noise levels of 15, 25, and 50.

Data Set	Algorithm	Sigma = 15	Sigma = 25	Sigma = 50
TNO	DnCNN	34.2508/0.9381	32.5658/0.9181	30.1155/0.8799
	xUnit	34.2516/0.9387	32.5917/0.9194	30.2127/0.8835
	ECNDNet	34.2549/0.9387	32.5721/0.9194	30.144/0.8832
	ADNet	**34.3319/0.9393**	32.6105/0.9200	30.2417/0.8852
	MSANet	34.2822/0.9389	32.5634/0.9189	30.0448/0.8795
	MSFLNet	34.3009/0.9392	**32.6394/0.9203**	**30.2829/0.8861**

The bold one in the table is the best indicator.

**Table 5:** The average value of PSNR and SSIM of different algorithms on the dataset on SIDD.

Data Set	DnCNN	xUnit	ECNDNet	ADNet	MSANet	MSFLNet
SIDD	37.66/0.939	34.77/0.886	28.85/0.668	36.44/0.910	38.557/**0.956**	**38.634**/0.953

The bold one in the table is the best indicator.

**Table 6:** The average value of PSNR and SSIM of different algorithms on the dataset on PolyU.

Data Set	DnCNN	xUnit	ECNDNet	ADNet	MSANet	MSFLNet
PolyU	36.85/0.962	37.00/0.964	35.86/0.940	36.80/0.954	35.81/0.967	**37.24/0.973**

The bold one in the table is the best indicator.

**Table 7:** The average value of PSNR and SSIM of different modules on the dataset on SIDD.

Data Set	Baseline Model	RG+Baseline	FL+Baseline	RG+FL1	RG+FL2	MSFLNet
SIDD	37.06/0.930	37.88/0.943	37.56/0.947	38.31/0.952	38.46/0.952	**38.63/0.953**

The bold one in the table is the best indicator.

**Table 8:** The total number of model parameters and model calculations for each algorithm.

Data Set	DnCNN	xUnit	ECNDNet	ADNet	MSANet	MSFLNet
FLOPs	7.1 G	4.1 G	6.6 G	6.7 G	27.1 G	7.3 G
Parameters	0.14 M	0.08 M	0.13 M	0.13 M	7.99 M	0.14 M

The bold one in the table is the best indicator.

## Data Availability

The public datasets are used in this study. No new data were created or analyzed. Data sharing is not applicable to this article. The SIDD datasets can be found here (http://www.cs.yorku.ca/~kamel/sidd/dataset.php, accessed on 23 August 2023). The PolyU datasets can be found here (https://gitcode.net/mirrors/csjunxu/PolyUDataset, accessed on 23 August 2023). The DIV2K datasets can be found here (https://cv.snu.ac.kr/research/EDSR/DIV2K.tar, accessed on 23 August 2023). The TNO datasets can be found (https://s3-eu-west-1.amazonaws.com/pfigshare-u-files/1475454/TNO_Image_Fusion_Dataset.zip, accessed on 23 August 2023).
